# Phytochemical andrographolide modulates NF-κB and JNK in human neuroblastoma SH-SY5Y cells, a cell model for Parkinson's disease

**DOI:** 10.1016/j.heliyon.2020.e04121

**Published:** 2020-06-09

**Authors:** Albert J. Ketterman, Jeerang Wongtrakul, Chonticha Saisawang

**Affiliations:** a Institute of Molecular Biosciences, Mahidol University, 25/25 Putthamonthol Road 4, Salaya, Nakhon Pathom, 73170, Thailand; b Center for Molecular and Cell Biology for Infectious Diseases, Research Institute for Health Sciences, Chiang Mai University, PO.BOX 80 CMU, Chiang Mai, 50200, Thailand

**Keywords:** Biotechnology, Proteins, Biochemistry, Molecular biology, Aging, Andrographolide, Traditional medicine, Oxidative stress, MAP kinase Signaling, NF-kB

## Abstract

*Andrographis paniculata* has been an important plant for traditional medicine in Asia for centuries. Andrographolide is the primary bioactive phytochemical from the plant and is known to exhibit many different protective effects through modulation of various proteins and signaling pathways. Andrographolide has been reported to exert anti-inflammatory and neuroprotective effects as well as being an antioxidant itself. We therefore studied whether andrographolide could provide protective effects to the SH-SY5Y neuroblastoma cell model for Parkinson's disease. In this study, we observed andrographolide inhibiting activation of NF-κB p65 (nuclear factor kappa-light-chain-enhancer of activated B cells) and JNK MAPK (c-Jun N-terminal Kinase Mitogen-Activated Protein Kinase) pathways, however, it did not provide any protective effect against induced stress in the SH-SY5Y cells. We propose the sustained low-level activation of JNK and the inhibition of NF-κB promoted ROS (Reactive Oxygen Species) production that yielded the observed cell death. Therefore, the protective effects observed with andrographolide appear to be cell/tissue specific responses.

## 1 Introduction

Parkinson's disease (PD) is the second most common movement disorder and one of the most common neurodegenerative diseases worldwide (Berg et al., 2007). PD is a progressive neurodegenerative disorder with the idiopathic symptoms resulting from the degeneration of dopaminergic neurons. The pathological hallmark of PD is the gradual loss of dopaminergic neurons of the substantia nigra pars compacta (SNpc) area in the brain leading to the depletion of dopamine (Xie et al., 2010). Loss of dopamine negatively affects the nerves and muscles controlling movement and coordination, resulting in the major symptoms characteristic of PD. These symptoms include differing extents of rigidity, bradykinesia, tremor, and dementia (de Lau and Breteler, 2006; Lees et al., 2009).

PD is a multifactorial disease with one of the salient mechanisms involved in the onset and progression being mitochondrial damage caused by oxidative stress (Lotharius and Brundin, 2002). The pathogenic mechanisms of sporadic PD have been difficult to elucidate as a range of factors are involved including environmental toxins, mitochondrial dysfunction and oxidative stress. Evidence suggests that the exposure of SNpc dopaminergic neurons to oxidative stress could extensively contribute to neurodegeneration in PD (Dagda et al., 2009; Lotharius and Brundin, 2002). As a consequence, increased oxidative damage which leads to the depletion of antioxidants such as glutathione (GSH), have been detected in PD brain (Lushchak, 2012). Oxidative stress is a deleterious circumstance that results from insufficient scavenging of reactive oxygen species (ROS) (Thannickal and Fanburg, 2000). In cells, exposure to ROS triggers a series of events including depletion of antioxidant defenses and oxidative modification of proteins, lipids, and nucleic acids.

*Andrographis paniculata* has been an important plant for traditional medicine in many Asian countries for centuries (Akbar, 2011). In Thailand, the Ministry of Public Health has listed this plant known as Fah Talai Jone on “The National List of Essential Drugs A.D. 1999” (List of Herbal Medicinal Products) (Jarukamjorn and Nemoto, 2008). Andrographolide is a bicyclic diterpene lactone and the primary bioactive phytochemical from the plant *Andrographis paniculata.* Andrographolide has been reported to exhibit antioxidant, immunomodulatory, antihyperglycemic, anti-inflammatory, antimicrobial, antiprotozoal, antiviral, anticancer, cardiovascular protection, hepatoprotective and neuroprotective effects (Akbar, 2011, Chen et al., 2009, Mishra et al., 2011, Singha et al., 2003, Wintachai et al., 2015). Its protection mechanisms involve several pathways including the inhibition of MAP kinase (Mitogen-Activated Protein Kinase) pathways, activation of NF-κB (nuclear factor kappa-light-chain-enhancer of activated B cells) and PI3K (phosphoinositide 3-kinase) pathways for anti-inflammatory responses. Andrographolide activates transcription; suppresses cyclins, cyclin-dependent kinases (CDKs), metalloproteinases, growth factors, heat shock proteins (hsp-90), and induces tumor suppressor proteins p53 and p21, which leads to inhibition of cancer cell proliferation, survival, metastasis, and angiogenesis (Chen et al., 2014, Islam, 2017). At present, evaluation of pharmacological activities have been carried out for several synthesized andrographolide derivatives but comprehensive studies on their neuroprotective roles remain minimal (Yan et al., 2013, Zhang et al., 2014).

In this study, we examined the antioxidant effect of andrographolide on the SH-SY5Y neuroblastoma cell model for Parkinson's disease. Under our experimental conditions we observed that pre-treatment of the cells with andrographolide does not ameliorate stress although it does inhibit the activation of the p65 subunit of NF-κB as well as the JNK MAPK signaling pathway.

## 2 Materials and methods

### 2.1 Chemicals and antibodies

Andrographolide (purity >99%) was purchased from Sigma-Aldrich. It was dissolved in 100% DMSO (dimethyl sulfoxide) and kept at -80 °C. Andrographolide was diluted to the final concentration of less than 0.1% of DMSO. Antibodies were obtained from Cell Signaling Technology including anti-phospho-Akt (Ser473) (D9E) XP® (#4060), anti-phospho-MEK1/2 (Ser217/221) (41G9) (#9154), anti-phospho-NF-κB p65 (Ser536) (93H1) (#3033), anti-phospho-SAPK/JNK (Thr183/Tyr185) (G9) (#9255) and anti-phospho-p44/42 MAPK (Erk1/2) (Thr202/Tyr204) (D13.14.4E) XP®. The following antibodies: anti-phospho-p38 MAPK (pThr180 + Tyr182) (S.417.1) (Thermo Fisher), anti-caspase-3 (BioVision), anti-tyrosine hydroxylase (TH, sc-25269) and anti-β tubulin (JDR.3B8) (Santa Cruz) were obtained from the stated respective companies.

### 2.2 Cell culture and treatment

SH-SY5Y cell line was purchased from ATCC and was maintained at 37 °C under 5% CO_2_ in DMEM-F12 media supplemented with 10% FBS and 100 units/ml of penicillin/streptomycin. Cells were grown on 60 mm dishes until they reached a density of 80% confluency and treated the following day with 10 μM andrographolide alone for 2 h, or 1 mM H_2_O_2_ for 15 min, or pre-treatment of andrographolide for 2 h prior to 1 mM H_2_O_2_ treatment for 15 min. Cells treated with 0.1% DMSO were used as control.

### 2.3 Cell viability assay

Cells were grown on 96-well plates at a density of 80% confluency in duplicates for 24 h prior to treatment. After cell treatments, 10 μl of 5 mg/ml MTT (3-(4,5-dimethylthiazol-2-yl)-2,5-diphenyltetrazolium bromide) reagent was added to each well and incubated for 4 h at 37 °C. The plates were centrifuged, media were removed, and cells were washed with PBS (phosphate buffered saline). 100 μl of DMSO was added to each well and further incubated for 15 min. Absorbance was measured at 490 nm in a microplate reader, SpectraMax 250.

### 2.4 Measurement of intracellular reactive oxygen species (ROS)

The intracellular ROS was monitored using the fluorescent probe 2′, 7′-dichlorofluorescin diacetate (DCFH-DA), which can be oxidized to the highly fluorescent compound dichlorofluorscein (DCF). Cells were grown on black 96-well plates for 24 h before treatment. After stimulation, cells were incubated with 10 μM DCFH-DA at 37 °C for 30 min and washed with PBS. The fluorescence intensity was measured using a fluorescence microplate reader (Beckman) with an excitation wavelength of 485 nm and an emission wavelength of 535 nm.

### 2.5 Immunoblotting

The cell samples were lysed in NP-40 lysis buffer (150 mM NaCl, 1% NP-40 and 50 mM Tris-HCl, pH 8.0) and with periodic vortexing while incubating on ice for 30 min. After that, samples were centrifuged at 10,000 rpm for 30 min. The supernatants were collected and protein concentration was measured using the Bradford method with BSA as protein standard. The immunoblotting was performed with a standard protocol using the appropriate antibodies. Briefly, 20–100 μg of proteins were used in SDS-PAGE and then transferred onto nitrocellulose membrane. The membranes were blocked with 5% skim milk or BSA in PBS-T (PBS buffer containing 0.2% Tween-20) buffer. The membranes were incubated with a dilution of 1:1000 fold of primary antibody overnight at 4 °C. After washing the membranes with PBS-T buffer, 1:10,000 fold dilution of appropriate secondary antibody was added to the membrane and incubated at room temperature for 3 h. The signals were visualized using Amersham ECL Prime Western Blotting Detection Reagent and captured by Azure cSeries CCD camera.

### 2.6 Statistical analysis

One-way analysis of variance (ANOVA) followed by Tukey's multiple comparisons test was performed using Prism 6 (GraphPad Software, Inc., San Diego, CA, USA) to evaluate statistical significance of differences. Data are presented as mean ± SEM of 3 independent experiments. Results are considered statistically significant at *p*-value < 0.05.

## 3 Results and discussion

### 3.1 Andrographolide protects cells by reducing ROS production

**Figure 1:**
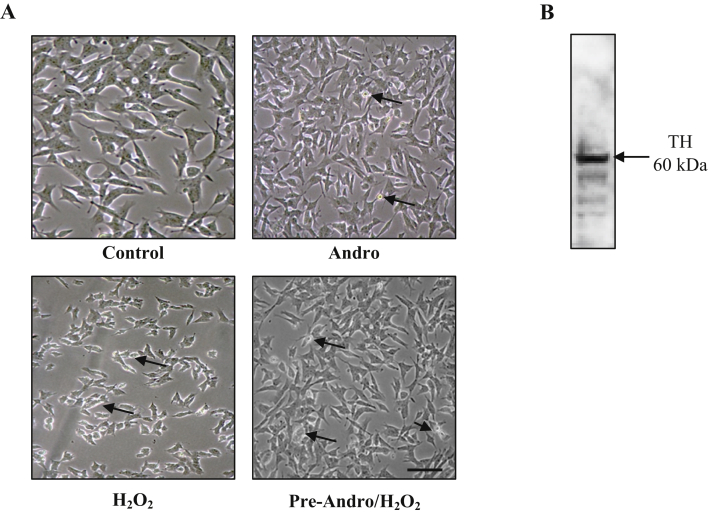
Cell morphology and dopaminergic phenotype. Cells were grown on 60 mm dishes until they reached a density of 80% confluency and treated the following day with 10 μM andrographolide alone for 2 h, or 1 mM H_2_O_2_ for 15 min or pre-treatment of andrographolide for 2 h prior to 1 mM H_2_O_2_ treatment for 15 min. Cells treated with 0.1% DMSO were used as control. (A) The morphology of the treated SH-SY5Y cells compared to control cells observed under inverted phase-contrast microscopy, scale bar = 50 μm. The arrows indicate the cells that have undergone morphological changes and died. (B) The expression of tyrosine hydroxylase (TH) in SH-SY5Y neuroblastoma cells was detected by western blot. The full non-adjusted western blot image of Figure 1B is shown in Supplementary Figure 1. The full non-adjusted western blot image of Figure 1B.

**Figure 2:**
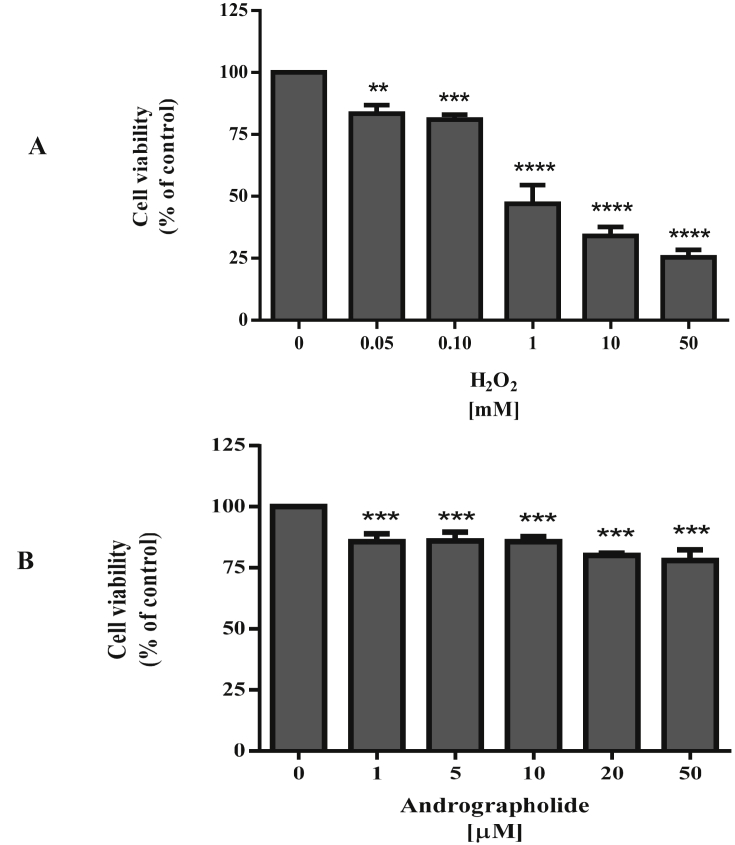
Effects of hydrogen peroxide and andrographolide on cell viability. (A) Cell viability (MTT) assay of SH-SY5Y cells exposed to H_2_O_2_ at the indicated concentrations for 15 min. (B) Cell viability (MTT) assay of SH-SY5Y cells exposed to andrographolide at the indicated concentrations for 2 h. The experiments were performed for at least 3 independent assays. Results are presented as mean ± SD. ∗*p* < 0.05, ∗∗*p* < 0.01,∗∗∗*p* < 0.001 and ∗∗∗∗*p* < 0.0001 vs control.

**Figure 3:**
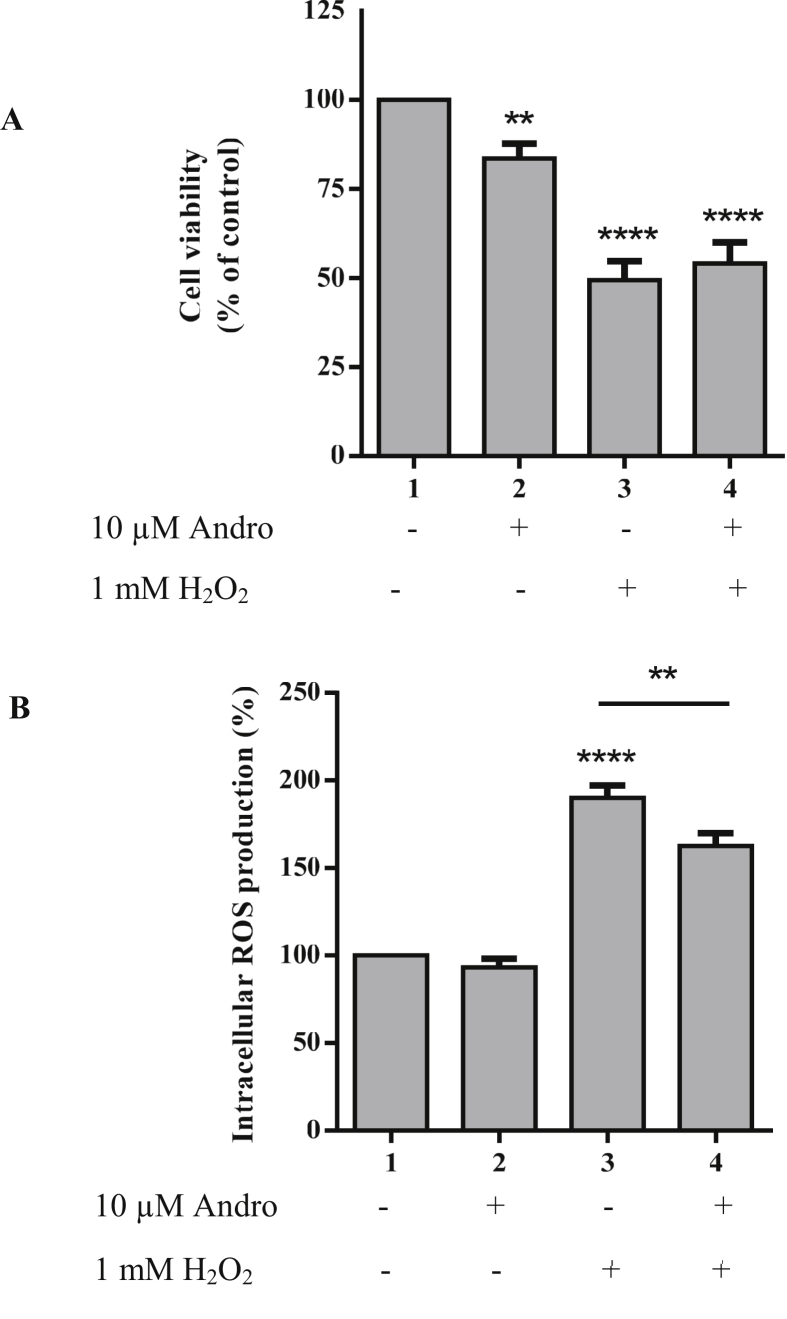
Effects of andrographolide on cell survival and ROS reduction. (A) The effect of the pre-treatment of andrographolide on cell viability. 10 μM of andrographolide was used to treat the cells prior to oxidative stress induction with 1 mM H_2_O_2_ for 15 min. (B) The same conditions as (A) but for the detection of ROS production. The experiments were performed for at least 3 independent assays. Results are presented as mean ± SD. ∗*p* < 0.05, ∗∗*p* < 0.01,∗∗∗*p* < 0.001 and ∗∗∗∗*p* < 0.0001 vs control.

There are many reports of the cytotoxicity of andrographolide although the extent seems to vary with cell type (see review and references therein (Islam et al., 2018)). To ensure our experimental conditions were not severely cytotoxic we obtained preliminary observations showing low cell death numbers and no change in cell morphology (Figure 1A). We also confirmed the dopaminergic phenotype of our SH-SY5Y cells with western blot detection of tyrosine hydroxylase (Figure 1B). Tyrosine hydroxylase is the initial and rate-limiting enzyme in dopamine synthesis (Cartier et al., 2010; Haycock and Haycock, 1991; Zigmond et al., 1989). This data validated our cells were of the dopaminergic phenotype and still mainly viable within the parameters of our experimental conditions. To examine the cytotoxicity of H_2_O_2_, SH-SY5Y cells were treated with varied concentrations of H_2_O_2_ and cell viability was determined using the MTT assay (Figure 2A). At 1 mM H_2_O_2_ treatment for 15 min, cell viability was 47 ± 7.6%, so this treatment condition was selected for subsequent experiments. Cytotoxicity of andrographolide to the cells was also investigated at 2 h treatment of 1, 5, 10, 20 and 50 μM (Figure 2B). Cell survival rate under these andrographolide concentrations was 78–86% which indicates that these conditions were not significantly toxic to the cells. To investigate whether andrographolide protects cells from oxidation, SH-SY5Y cells were pre-treated with 10 μM of andrographolide for 2 h prior to induction of intracellular ROS with 1 mM H_2_O_2_. The MTT assay was employed to evaluate the protective effect of andrographolide against oxidative stress (Figure 3A). Although andrographolide does not appear to increase cell viability of the pre-treated samples it does significantly reduce the ROS production as observed by a decreased DCF fluorescence intensity compared to the H_2_O_2_ only treated cells (Figure 3B).

### 3.2 Effects of andrographolide on signaling molecules

**Figure 4:**
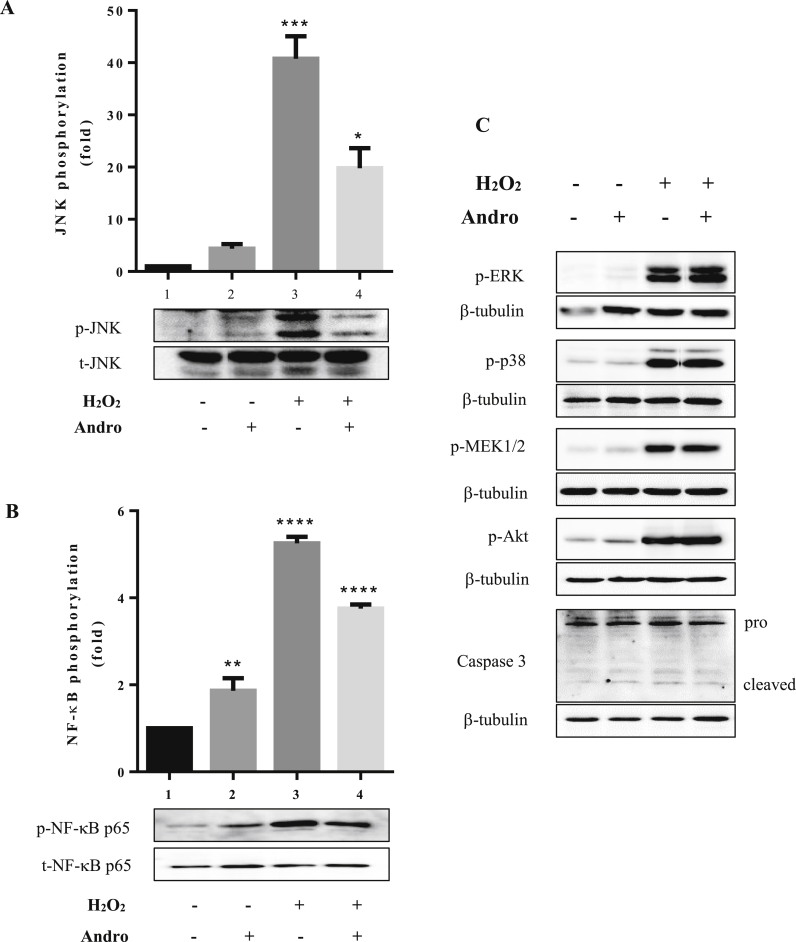
Effects of andrographolide on different signaling pathways. 20–100 μg of cell lysates were used for western blot analysis. Lane 1 is control cells treated with 0.1% DMSO. Lane 2 and 3 are cells treated with 10 μM of andrographolide for 2 h and 1 mM of H_2_O_2_ for 15 min alone, respectively. Lane 4 contains cells pre-treated with 10 μM andrographolide for 2 h and then treated with 1 mM of H_2_O_2_ for 15 min. The membranes were stripped and re-probed with A) anti-total JNK, B) total NF-κB p65 or C) anti-β-tubulin antibody for loading control. The band intensities for phosphorylation was quantitated by ImageJ program and bar graphs were plotted using the GraphPad prism 6 program. The quantitation was analyzed from 3 independent experiments using one-way analysis of variance (ANOVA) followed by Tukey's multiple comparisons test to compare the treated groups to the control. Data are expressed as mean ± SEM. Asterisks indicate significant differences at ∗*p* < 0.05, ∗∗*p* < 0.01,∗∗∗*p* < 0.001 and ∗∗∗∗*p* < 0.0001 when compared to untreated controls. For full non-adjusted western blot images of Figure 4 see Supplementary Figure 2. The full non-adjusted western blot images of Figure 4.

Several studies have demonstrated that the phosphorylation of MAPKs is mediated by andrographolide (Islam et al., 2018; Yan et al., 2013; Yen et al., 2016). To explore the protective effect of andrographolide on oxidative stress, 10 μM of andrographolide was used for pre-treatment of cells before generating oxidative stress with H_2_O_2_ (Figure 4). The cells that were treated with andrographolide alone showed a slight increase in the phosphorylation (activation) of JNK as well as the inflammatory NF-κB pathway (Figure 4A and 4B). H_2_O_2_ treated cells were used as a positive control. All 3 MAPKs (JNK, p38 MAPK and ERK1/2) were activated in response to oxidative stress under those conditions (Figure 4C). However, only the JNK pathway was shown to be affected by andrographolide pre-treatment with the phosphorylation signal decreased about 50% compared to the positive control (Figure 4A). Although JNK is known to be pro-apoptotic, it may play important roles in other forms of cell death, such as necrosis and autophagy. JNK has been shown to contribute to necrosis by promoting ROS production when the NF-κB pathway is inhibited (Ventura et al., 2004). The sustained late phase of JNK activation (1–6 h) appears to be regulated by ROS as well as inhibition of MAPK phosphatase activity which itself may be due to ROS surges (Ventura et al., 2006; Weston and Davis, 2007).

Under these experimental conditions we did not observe any protective effects from andrographolide pre-treatment which we think is a result of the prolonged activation of JNK. This result suggests that the consequences of the high H_2_O_2_ concentration were too great for the andrographolide response to overcome. With this in mind, we decreased the H_2_O_2_ to 0.1 mM for a 24 h treatment (Han et al., 2014). We observed similar results as with the higher H_2_O_2_ treatment (results not shown). Although there are reports concerning the protective effects of andrographolide, many appear to be analysis of patient samples or whole animal studies (reviewed in (Lu et al., 2019; Yang et al., 2017)). However, *in vitro* cell culture studies with various cell types suggest protective effects of andrographolide (for example (Chen et al., 2012; Islam et al., 2018; Li et al., 2007a, Li et al., 2007b; Yan et al., 2013)). The salient points of these studies are that the impact of andrographolide treatment varies with cell and tissue type studied, for example, NF-κB and JNK-MAPK pathways may be activated in some environments while other cell/tissue types show inhibition of these pathways. Consequently, although the andrographolide pre-treatment seems to modulate several signalling pathways, there appears to be no detectable protective effect for SH-SY5Y cells. Therefore, whether or not andrographolide, or a chemical derivative, can effectively be used therapeutically, remains a complex question and may only be solved with a targeted delivery system.

## Declarations

### Author contribution statement

C. Saisawang: Conceived and designed the experiments; Performed the experiments; Analyzed and interpreted the data; Wrote the paper.

J. Wongtrakul: Analyzed and interpreted the data.

A. Ketterman: Conceived and designed the experiments; Analyzed and interpreted the data; Wrote the paper.

### Funding statement

A. Ketterman and C. Saisawang were supported by 10.13039/501100004156Mahidol University.

### Competing interest statement

The authors declare no conflict of interest.

### Additional information

No additional information is available for this paper.

## Appendix A Supplementary data

Supplementary Material 1.docx

Supplementary Material 2.docx Supplementary Material 2.docx
