# Supplementary material for: Phytochemical andrographolide modulates NF-κB and JNK in human neuroblastoma SH-SY5Y cells, a cell model for Parkinson's disease
Source: Heliyon. 2020 Jun 9;6(6):e04121. doi: 10.1016/j.heliyon.2020.e04121 (PMC7287258; doi:10.1016/j.heliyon.2020.e04121)
Supplement: Supplementary file 1 — Supplementary Material 1.docx [file mmc1.docx]

**Supplementary Figure 1**

**H_2_O_2_  - - + +**

**Andro - + - +**

**
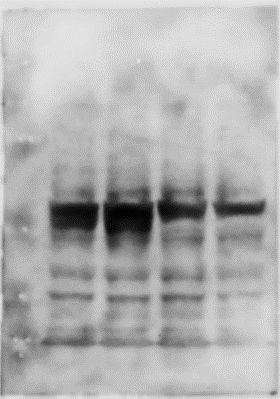
**

TH

60 kDa

**Supplementary Figure 1. The full non-adjusted western blot image of Figure 1B.** The expression of tyrosine hydroxylase (TH) in SH-SY5Y neuroblastoma cells was detected by western blot. Cells were grown on 60 mm dishes until they reached a density of 80% confluency and treated the following day with 10 µM andrographolide alone for 2 hr, or 1 mM H_2_O_2_ for 15 min or pre-treatment of andrographolide for 2 hr prior to 1 mM H_2_O_2_ treatment for 15 min. Cells treated with 0.1% DMSO were used as control.
