# Supplementary material for: Phytochemical andrographolide modulates NF-κB and JNK in human neuroblastoma SH-SY5Y cells, a cell model for Parkinson's disease
Source: Heliyon. 2020 Jun 9;6(6):e04121. doi: 10.1016/j.heliyon.2020.e04121 (PMC7287258; doi:10.1016/j.heliyon.2020.e04121)
Supplement: Supplementary Material 2.docx [file mmc2.docx]

**Supplementary Figure 2**

**H_2_O_2_  - - + +**

**Andro - + - +**

**H_2_O_2_  - - + +**

**Andro - + - +**


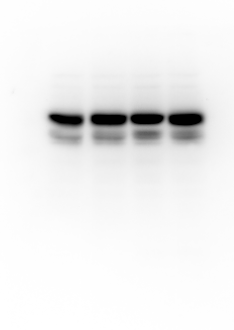


t-JNK

P46

P54

p-JNK


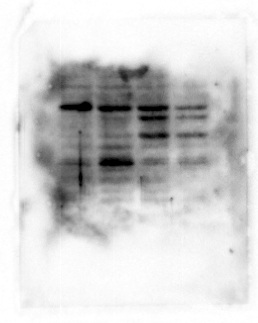


t-NF-κB


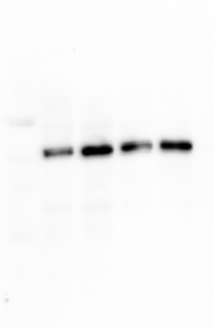


P65


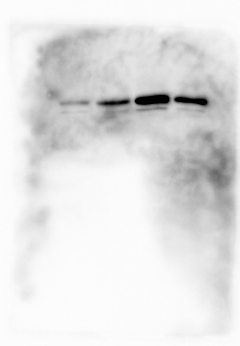


p-NF-κB

55 kda

Tubulin for ERK


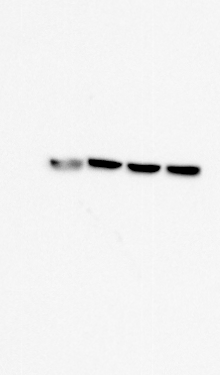


P44

P42

p-ERK


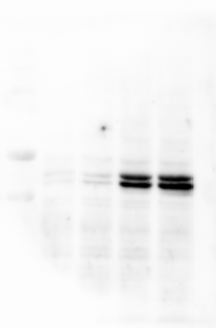


**H_2_O_2_  - - + +**

**Andro - + - +**

**H_2_O_2_  - - + +**

**Andro - + - +**


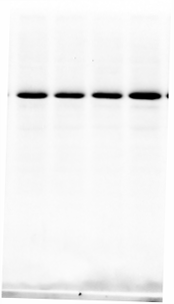


Tubulin for p38

55 kda


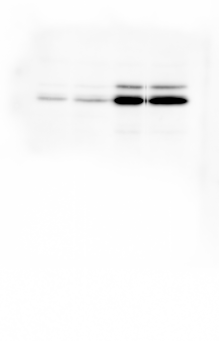


p-p38

P38


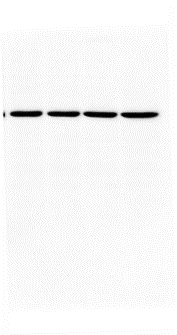


Tubulin for MEK1/2

55 kda

p-MEK


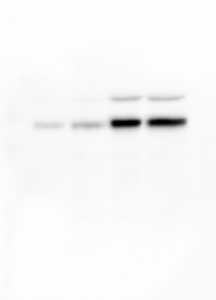


45 kda

Tubulin for Akt


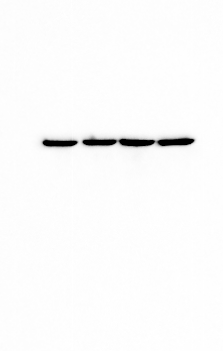


55 kda

p-Akt

p-Akt


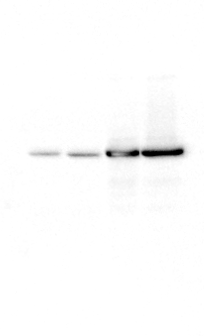


60 kda

Tubulin for caspase 3

55 kda


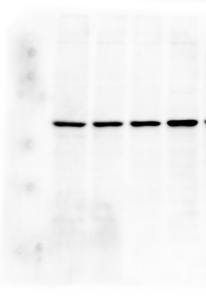


Caspase 3

35 kda

17 kda


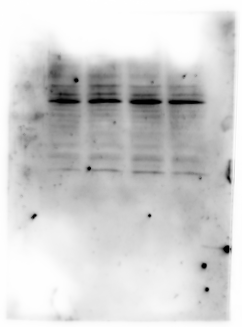


**Supplementary Figure 2. The full non-adjusted western blot images of Figure 4.** 20-100 μg of cell lysates were used for western blot analysis. Lane 1 is control cells treated with 0.1% DMSO. Lane 2 and 3 are cells treated with 10 μM of andrographolide for 2 hr and 1 mM of H_2_O_2_ for 15 min alone, respectively. Lane 4 contains cells pre-treated with 10 μM andrographolide for 2 hr and then treated with 1 mM of H_2_O_2_ for 15 min. The membranes were stripped and re-probed with A) anti-total JNK, B) total NF-κB p65 or C) anti-β-tubulin antibody for loading control. For analysis of the membranes see Figure 4 and its legend.
